# Voltage and Current Sensor Fault Diagnosis Method for Traction Converter with Two Stator Current Sensors

**DOI:** 10.3390/s22062355

**Published:** 2022-03-18

**Authors:** Hongwei Tao, Tao Peng, Chao Yang, Jinqiu Gao, Chunhua Yang, Weihua Gui

**Affiliations:** School of Automation, Central South University, Changsha 410083, China; hongwei.tao@csu.edu.cn (H.T.); chaoyang@csu.edu.cn (C.Y.); JinqiuG@csu.edu.cn (J.G.); ychh@csu.edu.cn (C.Y.); gwh@csu.edu.cn (W.G.)

**Keywords:** fault diagnosis, sensor fault, traction converter, hardware-in-the-loop (HIL) test

## Abstract

The traction converter is one of the key components of high-speed trains. Current and voltage sensor faults in the converter may lead to feedback values deviation and system degradation, which will bring security risks to the train. This paper proposes a real-time fault diagnosis method for grid current, DC-link voltage and stator current sensor faults in the traction converter with two stator current sensors, which can not only detect and locate faults but also identify the types of faults. Moreover, the faults considered in this paper are incipient. First, the DC-link model is established, and the fault is detected by the residual of the DC-link voltage. Next, the differential of DC-link voltage residual is calculated, which is applied to fault location. Then, according to the change of the differential values, different fault types are determined. Finally, the hardware-in-the-loop (HIL) platform is built and the effectiveness and accuracy of the proposed method are verified by the HIL tests.

## 1. Introduction

With the rapid development of high-speed trains, their safety has received increasing attention [1,2,3]. The traction converter is one of the most important components and also a main fault source of high-speed trains [4,5,6]. Because of the high requirements on train safety and reliability, the fault diagnosis methods of traction converter have been a research hotspot in recent years [7,8,9]. However, most of the studies focus on the power device fault; the sensors provide critical information to the traction control unit (TCU). A sensor fault will lead to the deviation of the corresponding feedback signal, which will degrade the control performance of the system and even cause secondary faults. It is important to diagnose the sensor fault in time.

The traction converter consists of a rectifier and a inverter. For the rectifier, there are some sensor fault diagnosis methods, which are for the grid current and DC-link voltage sensor faults. In [10], a model-based fault diagnosis method is proposed for the grid current fault; the signal prediction model is developed based on a data-driven method. However, the fault in the DC-link voltage is not considered, which will affect the fault diagnosis results. In [11], the fault diagnosis method can deal with sensor faults and open-circuit faults in the rectifier. Nonetheless, a additional voltage sensor is needed, and the sensor fault is ground fault, which may shut down the system before the fault is diagnosed. The faults in both the grid current and DC-link voltage sensors are included in [12], and the state observer-based method is applied to fault diagnosis. In [13], a sensor fault diagnosis and system reconfiguration approach is presented for the traction rectifier, and the method is based on sliding mode observer. However, the above two methods do not have the ability to distinguish the fault type. The studies on the inverter are more than the rectifier, which focus on stator current sensor faults [14,15,16]. Most of them require three stator current sensors. However, in practical application, in order to save costs, there are generally only two stator current sensors in the system, which increases the difficulty of the sensor fault diagnosis. In [17], the third-difference operator employed in the motor line current is used for fault detection of the line sensor faults. A fault detection method for sensor faults in electrical drives is proposed in [18], which is derived from a parity space approach and based on temporal redundancies. However, the two above methods can only used for fault detection and can not locate the faults. In [19], the imbalance current in the motor stator phases is used for fault diagnosis. However, if only two stator current sensors in the system and the fault is relatively incipient, the imbalance will not be obvious and difficult to be extract. In [20], a robust observer-based method is proposed and can diagnose the stator current sensor faults. Similarly, there are three sensors to measure the stator currents. A fault diagnosis method based on artificial neural network can deal with the current sensor faults is given in [21]. In [22], a data-driven method can identify the stator sensor fault types. The artificial intelligence algorithms or data-driven methods do not need circuit analysis or models which is suitable for complicated systems. Nevertheless, it requires large amounts of data and computational effort. Thus, it is not good a candidate for traction inverter fault diagnosis. A fault diagnosis method is proposed in [23], which is for three-phase inverters with two stator current sensors. A fault diagnosis strategy for the matrix converter is shown in [24], which only requires two current sensors. Although the rectifier and inverter are separated by the capacitor in the DC-link, they also influence each other. Especially for the voltage sensor fault, both the rectifier and inverter will be influenced obviously. So, a uniform method to the sensor faults in both the rectifier and inverter is necessary. In addition, only a few methods can diagnose the stator current sensor faults with two current sensors, and only a ground fault is considered. In practical applications, the offset and scaling faults are the common sensor faults [25,26]. There is a lack of a methods to diagnose the current and voltage sensor faults in the converter with two stator current sensors.

Motivated by the above discussions, a sensor fault diagnosis method is proposed in this paper, which can deal with the grid current, the DC-link voltage and stator current sensor faults, and only two stator current sensors are required. The residual of the DC-link voltage and the differentiation of the residual are calculated. The residual is applied to fault detection and the differentiation is used to determine the fault location and identify the fault type. There are three advantages of the proposed method. First, the incipient faults can be diagnosed, which can avoid further deterioration of faults. Second, the sensor faults in both the rectifier and inverter are taken into consideration. the interference between them is considered, which can reduce false alarms. Third, only two stator current sensor are required in process of fault diagnosis, which can save the cost and is more suitable for practical application. The paper is organized as follows. The topology of the traction converter is described and the DC-link model is established in Section 2. In Section 3, the faults are analyzed and the proposed fault diagnosis method is explain in detail. The HIL platform is established and the test results are shown in Section 4 and the conclusion is given in Section 5.

## 2. Converter Topology and DC-link Model

### 2.1. Converter Topology

**Figure 1:**
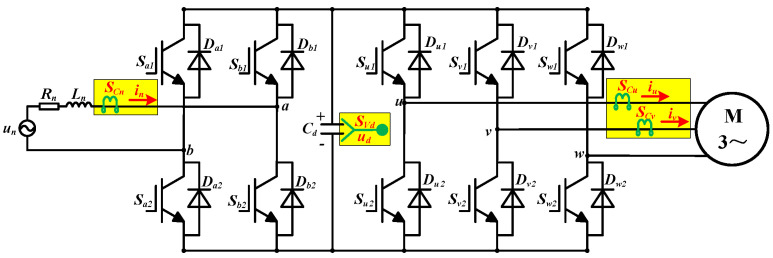
The topology of the two-level converter.

The topology of the two-level converter is shown in Figure 1, which is used to power the traction motor. There are five legs with the same structure. Every leg has two transistors and two freewheeling diodes, they are $S_{x1}$, $S_{x2}$, $D_{x1}$, and $D_{x2}$, respectively, *x* can be *a*, *b*, *u*, *v*, and *w*. $R_{n}$, and $L_{n}$ are the traction winding leakage resistance and inductance. $u_{n}$ is the grid voltage and $i_{n}$ is the grid current. $C_{d}$ is the capacitor in the DC-link, $u_{d}$ is the DC-link voltage. $i_{u}$ and $i_{v}$ are the stator currents of the traction motor. There are a grid current sensor, a voltage sensor and two stator current sensors in the converter. Another stator current is $i_{w}$, which is equal to the opposite of the sum of $i_{u}$ and $i_{v}$. $S_{Cn}$ is the grid current sensor, $S_{Vd}$ is the voltage sensor in the DC-link, $S_{Cu}$ and $S_{Cv}$ are the stator current sensors.

**Figure 2:**
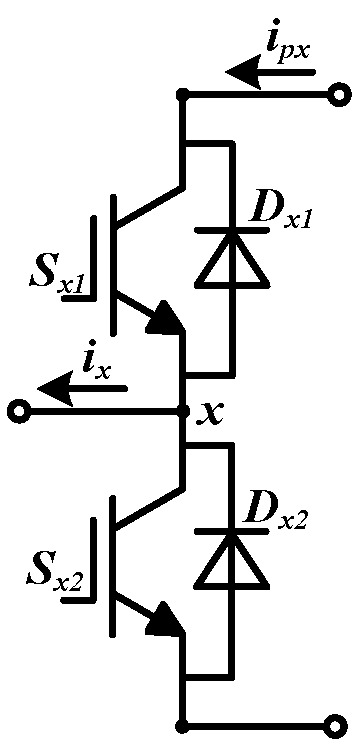
The topology of a single leg.

Figure 2 is the topology of a single leg in the converter. $i_{px}$ is the current from the DC-link to the leg and $i_{x}$ is the current from the leg to the grid side or traction motor.

### 2.2. DC-Link Model

**Figure 3:**
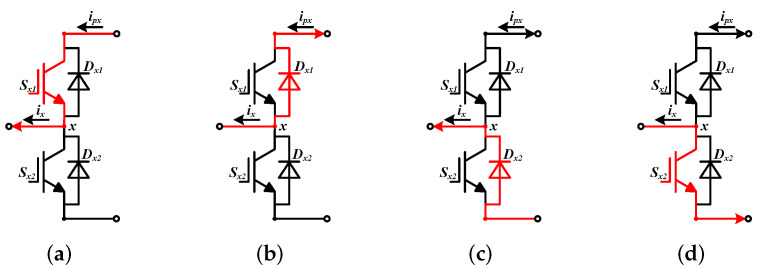
The current paths in a single leg. (**a**) The current flows through $S_{x1}$. (**b**) The current flows through $D_{x1}$. (**c**) The current flows through $D_{x2}$. (**d**) The current flows through $S_{x2}$.

Define $s_{x1}$ and $s_{x2}$ are the command signals of $S_{x1}$ and $S_{x2}$, respectively. The values of $s_{x1}$ and $s_{x2}$ can be 0 or 1. There are two kind of command signals of the leg, they are 10 and 01. When the command signals is 10, if $i_{x}\geq 0$, the current path is $S_{x1}$, which is shown in Figure 3a, $i_{px}$ is equal to $i_{x}$. If $i_{x}<0$, the current flows through $D_{x1}$, which is shown in Figure 3b, $i_{px}$ is equal to $i_{x}$ as well, while the command signals is 01, if $i_{x}\geq 0$, the current path is $D_{x2}$, it is given in Figure 3c, $i_{px}$ is zero. If $i_{x}<0$, the current flows through $S_{x2}$, it is shown in Figure 3d, $i_{px}$ is zero too.

According to the above analysis, the relationship between $i_{x}$ and $i_{px}$ is given as follows:

(1) $$i_{px}=s_{x1}i_{x}$$

Based on Kirchhoff’s current law. The DC-link model is established as follows:

(2) $$\frac{du_{d}}{dt}=-\frac{\sum_{x=a,b,u,v,w}s_{x1}i_{x}}{C_{d}}$$

## 3. Fault Analysis and Diagnosis

### 3.1. Sensor Fault Analysis

The offset fault and scaling fault are the common faults in the sensors. The offset fault is a superimposed value on the actual value, which is described in (3). The scaling fault means nonideal scaling gain of the actual value, it is shown in (4).

(3) $$yS=S+S_{off}$$

where *S* is the actual value and yS is the measured value of sensor. $S_{off}$ is the superimposed value caused by the offset fault.

(4) $$yS=K_{sca}S$$

where $K_{sca}$ is the nonideal scaling gain caused by the scaling fault.

In this paper, the offset and scaling faults in the grid current sensor and stator current sensors are taken into consideration. For the voltage sensor in the DC-link, the measured value is a DC variable, the effects of the offset and scaling faults are the same. So, only the offset fault is considered.

When an sensor fault occurs, the relationship between the measured value and actual value are given as follows:

(5) $$\left\{\begin{array}{c} yu_{d}=u_{d}+fS_{Vd}\\ yi_{\delta }=i_{\delta }+fS_{C\delta } \end{array}\right.$$

where δ can be *n*, *u*, and *v*. $yu_{d}$ and $yi_{\delta }$ is the feedback values of $u_{d}$ and $i_{\delta }$, respectively. $fS_{Vd}$ and $fS_{C\delta }$ are the faults occur in the sensors. In normal condition, $fS_{Vd}$ and $fS_{C\delta }$ are zero, while the sensor is faulty, the corresponding value is not zero anymore.

### 3.2. Fault Detection

In order to save cost, there are only two current sensors to measure stator currents, $yi_{u}$ and $yi_{v}$ can be measured by the sensor. Since the sum of three stator currents is zero, $yi_{w}$ can be calculated by:

(6) $$yi_{w}=-\left(yi_{u}+yi_{v}\right)$$

There is only one current sensor to measure $i_{n}$ in the grid side, based on the circuit topology, the relationship between $yi_{n}$ and $yi_{a}$, $yi_{b}$ is given as follows:

(7) $$\left\{\begin{array}{c} yi_{a}=-yi_{n}\\ yi_{b}=yi_{n} \end{array}\right.$$

Use the feedback values of sensors and (2), the estimated value of $u_{d}$ can be obtained as follows:

(8) $${\hat{u}}_{d}=-\int \frac{\sum_{x=a,b,u,v,w}s_{x1}yi_{x}}{C_{d}}dt$$

Then, the residual of $u_{d}$ can be given by:

(9) $${\tilde{u}}_{d}=yu_{d}-{\hat{u}}_{d}=yu_{d}+\int \frac{\sum_{x=a,b,u,v,w}s_{x1}yi_{x}}{C_{d}}dt$$

Since ${\tilde{u}}_{d}$ is obtained by integrator, there may be initial estimation errors and accumulative errors. The residual can not be applied to fault diagnosis directly. The differential of ${\tilde{u}}_{d}$ is described as follows:

(10) $$\frac{d{\tilde{u}}_{d}}{dt}=\frac{dyu_{d}}{dt}+\frac{\sum_{x=a,b,u,v,w}s_{x1}yi_{x}}{C_{d}}$$

Furthermore, (10) is discretized and given by:

(11) $$\frac{{\tilde{u}}_{d}\left(k\right)-{\tilde{u}}_{d}\left(k-1\right)}{\tau }=\frac{yu_{d}\left(k\right)-yu_{d}\left(k-1\right)}{\tau }+\frac{\sum_{x=a,b,u,v,w}s_{x1}\left(k-1\right)yi_{x}\left(k-1\right)}{C_{d}}$$

where τ is the sampling period.

Equation (2) is discretized and given by:

(12) $$\frac{u_{d}\left(k\right)-u_{d}\left(k-1\right)}{\tau }=-\frac{\sum_{x=a,b,u,v,w}s_{x1}\left(k-1\right)yi_{x}\left(k-1\right)}{C_{d}}$$

In normal condition, $yu_{d}\approx u_{d}$, combining (11) and (12), ${\tilde{u}}_{d}\left(k\right)\approx {\tilde{u}}_{d}\left(k-1\right)$. In order to avoid initial estimation errors and accumulative errors, the residual of $u_{d}$ is reconstructed. The new residual is shown as follows:

(13) $${\tilde{u}}_{d}^{{}^{\prime }}\left(k\right)=\left\{\begin{array}{cc} 0, & k=1\\ {\tilde{u}}_{d}^{{}^{\prime }}\left(k-1\right), & k\geq 2,|\frac{{\tilde{u}}_{d}\left(k\right)-{\tilde{u}}_{d}\left(k-1\right)}{\tau }|\leq h_{0}\\ {\tilde{u}}_{d}^{{}^{\prime }}\left(k-1\right)+{\tilde{u}}_{d}\left(k\right)-{\tilde{u}}_{d}\left(k-1\right), & k\geq 2,|\frac{{\tilde{u}}_{d}\left(k\right)-{\tilde{u}}_{d}\left(k-1\right)}{\tau }|>h_{0} \end{array}\right.$$

where ${\tilde{u}}_{d}^{{}^{\prime }}$ is the reconstructed voltage residual. $h_{0}$ is the threshold for residual accumulation.

If there is no fault in the system, ${\tilde{u}}_{d}\left(k\right)-{\tilde{u}}_{d}\left(k-1\right)$ is caused by the parameter or measurement errors, which is relatively small, while a sensor fault occurs, ${\tilde{u}}_{d}\left(k\right)-{\tilde{u}}_{d}\left(k-1\right)$ is mainly caused by the fault, which is much bigger. Thus, $h_{0}$ is easy to determine.

It can be seen that ${\tilde{u}}_{d}^{{}^{\prime }}$ can be influenced by all the measured values of sensors, so all the sensor faults can cause the changes of it. ${\tilde{u}}_{d}^{{}^{\prime }}$ can be applied to fault detection. The fault is detected by:

(14) $$F_{D}=\left\{\begin{array}{cc} 0, & |{\tilde{u}}_{d}^{{}^{\prime }}|\leq h_{1}\\ 1, & |{\tilde{u}}_{d}^{{}^{\prime }}|>h_{1} \end{array}\right.$$

where $h_{1}$ is the threshold of $|{\tilde{u}}_{d}^{{}^{\prime }}|$. $F_{D}$ is the fault detection function. If there is no fault in the system, $F_{D}=0$, while a fault occurs, $F_{D}=1$.

### 3.3. Fault Diagnosis

After the fault is detected, the fault should be located. When a fault occurs in $S_{Vd}$, the differential of ${\tilde{u}}_{d}^{{}^{\prime }}$ is shown as follows:

(15) $$\frac{d{\tilde{u}}_{d}^{{}^{\prime }}}{dt}=\frac{dfS_{Vd}}{dt}$$

When an offset fault occurs in $S_{Vd}$, $fS_{Vd}$ is a constant value, so $\frac{d{\tilde{u}}_{d}^{{}^{\prime }}}{dt}$ will increase only at the instant of fault occurrence. Then, $\frac{d{\tilde{u}}_{d}^{{}^{\prime }}}{dt}$ will be zero all the time.

When a fault occurs in $S_{Cn}$, the value of $\frac{d{\tilde{u}}_{d}^{{}^{\prime }}}{dt}$ is given by:

(16) $$\frac{d{\tilde{u}}_{d}^{{}^{\prime }}}{dt}=\frac{\left(s_{b1}-s_{a1}\right)fS_{Cn}}{C_{d}}$$

When a fault occurs in $S_{Cu}$, the value of $\frac{d{\tilde{u}}_{d}^{{}^{\prime }}}{dt}$ is calculated by:

(17) $$\frac{d{\tilde{u}}_{d}^{{}^{\prime }}}{dt}=\frac{\left(s_{u1}-s_{w1}\right)fS_{Cu}}{C_{d}}$$

In the same way, while $S_{Cv}$ is faulty, $\frac{d{\tilde{u}}_{d}^{{}^{\prime }}}{dt}$ is obtained as follows:

(18) $$\frac{d{\tilde{u}}_{d}^{{}^{\prime }}}{dt}=\frac{\left(s_{v1}-s_{w1}\right)fS_{Cv}}{C_{d}}$$

The differential of ${\tilde{u}}_{d}^{{}^{\prime }}$ is shown in (15)∼(18). After the fault occurs in $S_{Vd}$, the value is zero, while the fault in the current sensor, the value changes with the change of command signals. Thus, the fault can be located by:

(19) $$F_{Vd}=\left\{\begin{array}{cc} 0, & \mathrm{other}\\ 1, & |{\tilde{u}}_{d}^{{}^{\prime }}|>h_{1}⋂\left|\frac{d{\tilde{u}}_{d}^{{}^{\prime }}}{dt}\right|\leq h_{2} \end{array}\right.$$

(20) $$F_{Cn}=\left\{\begin{array}{cc} 0, & \mathrm{other}\\ 1, & \mathrm{if}\left\{\begin{array}{c} |\frac{d{\tilde{u}}_{d}^{{}^{\prime }}}{dt}|>h_{2},\left|s_{b1}-s_{a1}\right|=1\\ |\frac{d{\tilde{u}}_{d}^{{}^{\prime }}}{dt}|\leq h_{2},s_{b1}-s_{a1}=0 \end{array}\right. \end{array}\right.$$

(21) $$F_{Cu}=\left\{\begin{array}{cc} 0, & \mathrm{other}\\ 1, & \mathrm{if}\left\{\begin{array}{c} |\frac{d{\tilde{u}}_{d}^{{}^{\prime }}}{dt}|>h_{2},\left|s_{u1}-s_{w1}\right|=1\\ |\frac{d{\tilde{u}}_{d}^{{}^{\prime }}}{dt}|\leq h_{2},s_{u1}-s_{w1}=0 \end{array}\right. \end{array}\right.$$

(22) $$F_{Cv}=\left\{\begin{array}{cc} 0, & \mathrm{other}\\ 1, & \mathrm{if}\left\{\begin{array}{c} |\frac{d{\tilde{u}}_{d}^{{}^{\prime }}}{dt}|>h_{2},\left|s_{v1}-s_{w1}\right|=1\\ |\frac{d{\tilde{u}}_{d}^{{}^{\prime }}}{dt}|\leq h_{2},s_{v1}-s_{w1}=0 \end{array}\right. \end{array}\right.$$

where $h_{2}$ is the threshold of $|\frac{d{\tilde{u}}_{d}^{{}^{\prime }}}{dt}|$. $h_{1}$ and $h_{2}$ are obtained through a large number of tests, which are a tradeoff between the detection speed and accuracy. $fS_{Vd}$, $fS_{Cn}$, $fS_{Cu}$, and $fS_{Cv}$ are located by $F_{Vd}$, $F_{Cn}$, $F_{Cu}$, and $F_{Cv}$, respectively.

It should be noted that two of $s_{b1}-s_{a1}$, $s_{u1}-s_{w1}$, and $s_{v1}-s_{w1}$ may not be zero at the same time, but they can not be the same or opposite all the time. Hence, the current sensor fault can be distinguished by (19)∼(21). For current sensors, there are two kind of faults in them. After the fault is located, the fault type should be determined. Take the fault in $S_{Cn}$ as an example, the fault type is determined by the following method.

When an offset fault occurs in $S_{Cn}$, $fS_{Cn}$ is equal to ${\left(i_{n}\right)}_{off}$, which is a constant value. Therefore, $\frac{d{\tilde{u}}_{d}^{{}^{\prime }}}{dt}$ do not change obviously when $|s_{b1}-s_{a1}|=1$, while the fault is a scaling fault, $fS_{Cn}=K_{sca}i_{n}$. $i_{n}$ changes periodically, so $\frac{d{\tilde{u}}_{d}^{{}^{\prime }}}{dt}$ will change periodically as well. In half a current period, $|\frac{d{\tilde{u}}_{d}^{{}^{\prime }}}{dt}|$ will have values less than $h_{2}$. This feature can be used to distinguish the two types of faults. The fault types are determined by:

(23) $$F_{Cntype}=\left\{\begin{array}{cc} 1, & |\frac{d{\tilde{u}}_{d}^{{}^{\prime }}}{dt}{|}_{min}>h_{2},\left|s_{b1}-s_{a1}\right|=1\\ 2, & |\frac{d{\tilde{u}}_{d}^{{}^{\prime }}}{dt}{|}_{min}\leq h_{2},\left|s_{b1}-s_{a1}\right|=1 \end{array}\right.$$

where $|\frac{d{\tilde{u}}_{d}^{{}^{\prime }}}{dt}{|}_{min}$ is the minimum value of $|\frac{d{\tilde{u}}_{d}^{{}^{\prime }}}{dt}|$ in half a current period. $F_{Cntype}$ is the flag of fault types. When $F_{Cntype}=1$, the fault is an offset fault, while $F_{Cntype}=2$, there is a scaling fault in $S_{Cn}$.

Similarly, $F_{Cutype}$ and $F_{Cvtype}$ can be calculated. Based on the above method, the fault diagnosis rules are summarized in Table 1.

## 4. HIL Results

**Figure 4:**
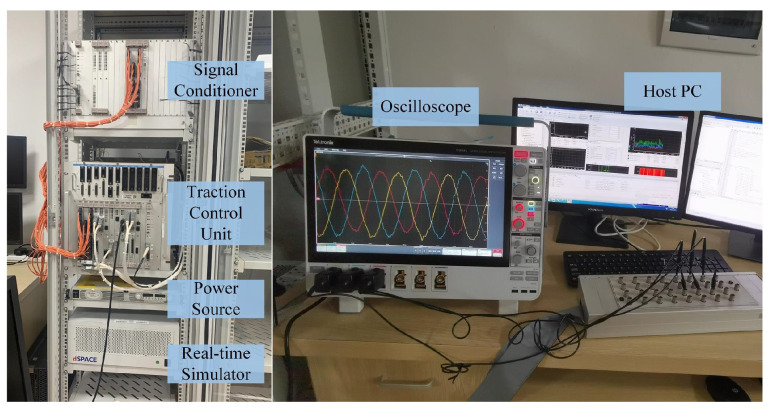
The HIL test platform.

Due to the danger which might be caused by the faulty converter and the high-voltage tests are hard to be implemented in the laboratory, the HIL tests are used to verify the correctness of the proposed fault diagnosis method. The HIL test platform is shown in Figure 4. It mainly includes a physical TCU, which is used in electrical multiple units (EMU), a real-time simulator, which is a dSPACE, and a host PC. Moreover, the power source provides power to the platform. The signal conditioner are used for voltage conversion between the TCU and real-time simulator. The oscilloscope are applied to the display of HIL results. The real-time simulator includes the 5203 board, which is an embedded Xilinx Kintex 7 field programmable gate array. The sampling period of the real-time simulator is 10 ns. The traction converter, the motor are emulated components. Both of them and the proposed fault diagnosis method are realized in the real-time simulator. The TCU is the physical component, the sampling period of it is 40 μs. The control algorithm is realized in the TCU, which receives the feedback signals from the real-time simulator and sends command signals to the real-time simulator. The HIL tests are controlled by the host PC. The traction converter and motor are used in CRH3 EMU and the main parameters are given in Table 2.

**Figure 5:**
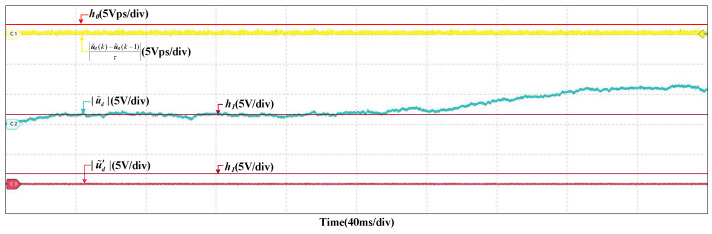
Orignal residual and reconstructed residuals of DC-link voltage in normal condition.

The residual of the DC-link voltage used in the HIL tests is the reconstructed one. In normal conditions, there will be initial estimation errors and accumulative errors. However, $|\frac{{\tilde{u}}_{d}\left(k\right)-{\tilde{u}}_{d}\left(k-1\right)}{\tau }|$ is less than $h_{0}$, the reconstructed residual will not be influenced. The original and reconstructed residual are given in Figure 5. It can be seen that the original residual increases and will exceed the $h_{1}$, but the reconstructed one does not change obviously and is less than $h_{1}$.

**Figure 6:**
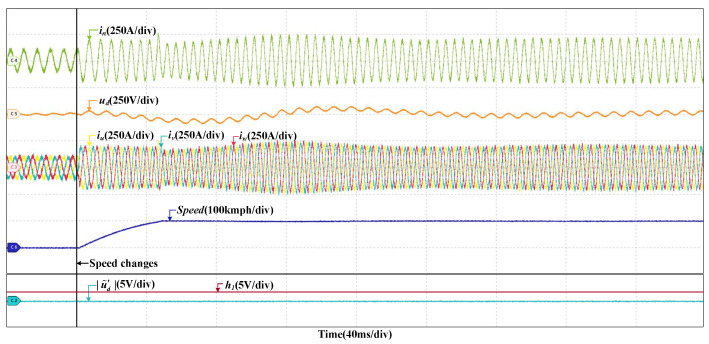
HIL results when the train speed changes.

**Figure 7:**
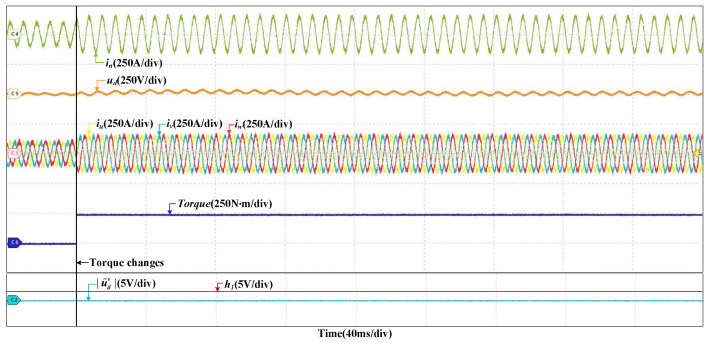
HIL results when the load torque changes.

When the train speed changes from 200 km/h to 300 km/h, the grid current, the DC-link voltage and the stator currents will change as well, but $|{\tilde{u}}_{d}^{{}^{\prime }}|$ is nearly zero and hardly changes; thus, no misdiagnosis occurs. The HIL results are shown in Figure 6. It can prove that the proposed method is not be affected by the change of train speed. When the load torque changes, the grid current and stator currents increase and there is larger fluctuation in the DC-link voltage, but $|{\tilde{u}}_{d}^{{}^{\prime }}|$ do not change, so obviously there are no false alarms. The HIL results are given in Figure 7. It shows that the proposed method is robust to load torque change.

### 4.1. Fault in DC-Link Voltage Sensor

**Figure 8:**
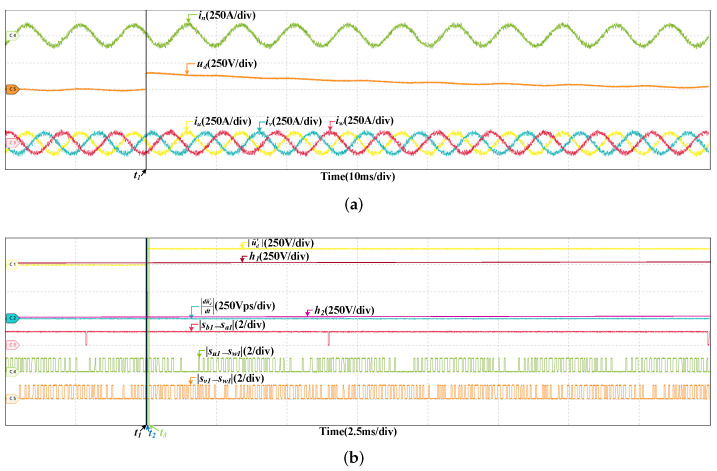
HIL results when the offset fault occurs in $S_{Vd}$ at 200 km/h. (**a**) The results of the grid current, DC-link voltage and stator currents. (**b**) The results of fault diagnosis.

**Figure 9:**
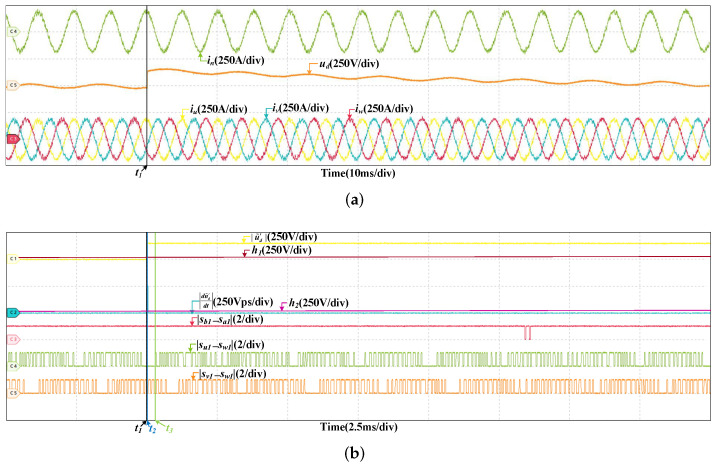
HIL results when the offset fault occurs in $S_{Vd}$ at 300 km/h. (**a**) The results of the grid current, DC-link voltage and stator currents. (**b**) The results of fault diagnosis.

To verify the robustness of the proposed fault diagnosis method, the HIL tests are curried out at two different speeds. One is 200 km/h, and the other is 300 km/h. Figure 8 are the HIL results when the offset fault occurs in $S_{Vd}$ at 200 km/h. The offset value is 150 V, which is about five percent of $u_{d}$. The fault occurs at $t_{1}$. It can be found that the grid current and three stator currents do not change obviously, but $u_{d}$ increases immediately. $|{\tilde{u}}_{d}^{{}^{\prime }}|$ grows rapidly, surpassing $h_{1}$ almost instantaneously, the fault is detected at $t_{2}$. Then, there are instants that the values of $|s_{b1}-s_{a1}|$, $|s_{u1}-s_{w1}|$, and $|s_{v1}-s_{w1}|$ are 1 but $|\frac{d{\tilde{u}}_{d}^{\prime }}{dt}|$ always less than $h_{2}$. If the fault occurs in $S_{Cn}$, when $|s_{b1}-s_{a1}|$ is 1, $|\frac{d{\tilde{u}}_{d}^{\prime }}{dt}|$ will larger than $h_{2}$. So the fault does not occurs in $S_{Cn}$. Similarly, it can be proved that the fault does not occur in $S_{Cu}$ and $S_{Cv}$. The fault is in $S_{Vd}$ and located at $t_{3}$. The HIL result at 300 km/h is shown in Figure 9, the offset values is also 150 V. The gird current and three stator currents are larger, and the DC-link voltage fluctuates more, but the fault diagnosis process is similar and the fault can be diagnosed within a short time. The instants of fault occurrence, fault detected and fault located are $t_{1}$, $t_{2}$, and $t_{3}$, respectively.

### 4.2. Fault in Grid Current Sensor

**Figure 10:**
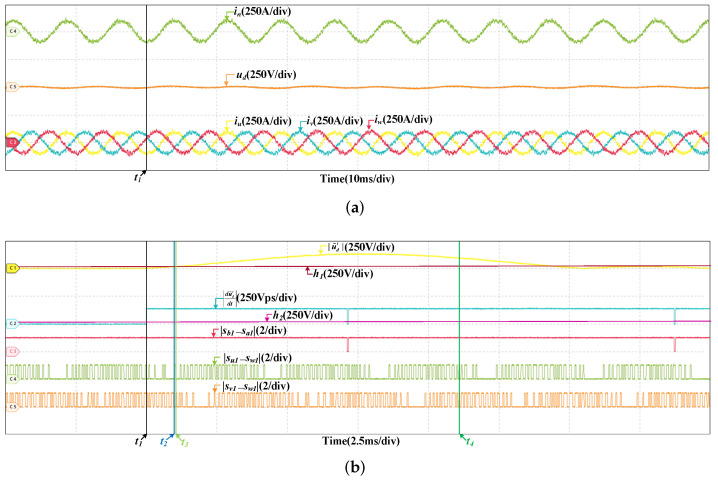
HIL results when the offset fault occurs in $S_{Cn}$ at 200 km/h. (**a**) The results of the grid current, DC-link voltage and stator currents. (**b**) The results of fault diagnosis.

**Figure 11:**
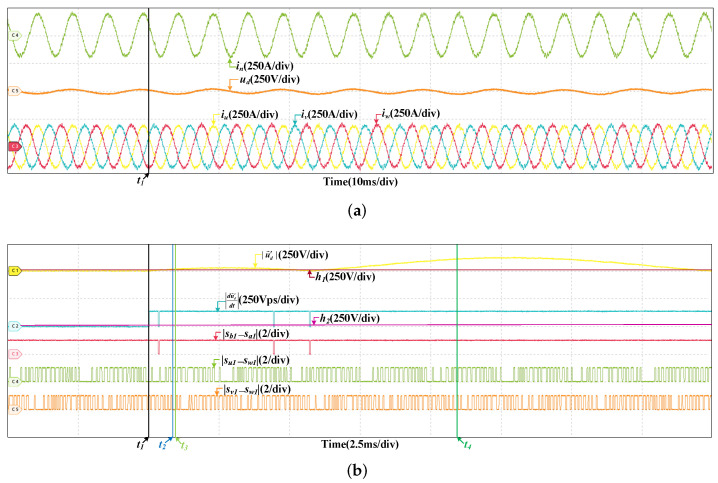
HIL results when the offset fault occurs in $S_{Cn}$ at 300 km/h. (**a**) The results of the grid current, DC-link voltage and stator currents. (**b**) The results of fault diagnosis.

There are two types of faults are considered in the grid current sensor. They are offset fault and scaling fault, so the fault type should be determined in the process of fault diagnosis. Figure 10 are the HIL results of the offset fault in $S_{Cn}$ at 200 km/h, and the offset value is 10 A. Since the fault is incipient and the effect of closed loop regulation, the currents and voltage of the system are not affected much by the fault. However, there are security risks in the system, the fault may deteriorate at any time. Thus, it should be diagnosed in time. After the fault occurs, $|{\tilde{u}}_{d}^{{}^{\prime }}|$ is influenced, it increases and surpasses $h_{1}$ quickly, the fault is detected. It can be seen that when $|s_{b1}-s_{a1}|=1$, $|\frac{d{\tilde{u}}_{d}^{{}^{\prime }}}{dt}|$ is larger than $h_{2}$. When $|s_{u1}-s_{w1}|=0$ or $|s_{u1}-s_{w1}|=0$, $|\frac{d{\tilde{u}}_{d}^{{}^{\prime }}}{dt}|$ is larger than $h_{2}$ as well. If the fault is in $S_{Vd}$, $|\frac{d{\tilde{u}}_{d}^{{}^{\prime }}}{dt}|$ will always be less than $h_{2}$, while a fault occurs in $S_{Cu}$ or $S_{Cv}$, $|\frac{d{\tilde{u}}_{d}^{{}^{\prime }}}{dt}|$ will be less than $h_{2}$ when $|s_{u1}-s_{w1}|=0$ or $|s_{u1}-s_{w1}|=0$. So the fault is occurs in $S_{Cn}$. Then, the fault is located. Then, in half a current period, there are no instants that $|\frac{d{\tilde{u}}_{d}^{{}^{\prime }}}{dt}|$ is less than $h_{2}$ when $|s_{b1}-s_{a1}|=1$. If the fault is a scaling fault, $|\frac{d{\tilde{u}}_{d}^{{}^{\prime }}}{dt}|$ changes periodically, there will be instants that $|\frac{d{\tilde{u}}_{d}^{{}^{\prime }}}{dt}|$ is less than $h_{2}$ when $|s_{b1}-s_{a1}|=1$ in half a current period. So the fault type is determined, which is an offset fault. Figure 11 are the results at 300 km/h, and the offset value is 10 A too. The fault diagnosis process is similar and omitted. The instants of fault occurrence, fault detected, fault located and fault diagnosed are $t_{1}$, $t_{2}$, $t_{3}$, and $t_{4}$, respectively. In the HIL results below, the meanings of $t_{1}$, $t_{2}$, $t_{3}$, and $t_{4}$ are same to them in Figure 10, and not explained again. In this paper, the current sensor faults are incipient and the influences on the currents and voltage are not obvious, the grid current, the DC-link voltage and three stator currents have little different when the offset and scaling faults in different location. So, the currents and voltage results are similar to the results in Figure 10 (200 km/h) and Figure 11 (300 km/h). The results below do not include the information of currents and voltage.

**Figure 12:**
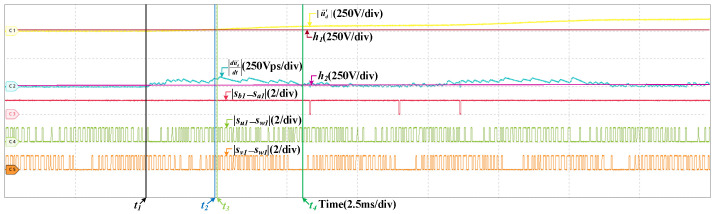
HIL results when the scaling fault occurs in $S_{Cn}$ at 200 km/h.

**Figure 13:**
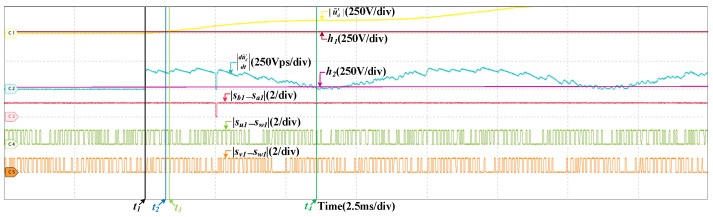
HIL results when the scaling fault occurs in $S_{Cn}$ at 300 km/h.

When the scaling fault occurs in $S_{Cn}$, the results are given in Figure 12, the train speed is 200 km/h and the scaling factor is 1.05. After the fault occurs, $|{\tilde{u}}_{d}^{{}^{\prime }}|$ increases and exceeds the threshold, the fault is detected, which is analogous to the offset fault. However, $|\frac{d{\tilde{u}}_{d}^{{}^{\prime }}}{dt}|$ is different, it changes periodically. $|\frac{d{\tilde{u}}_{d}^{{}^{\prime }}}{dt}|$ is related to $|s_{b1}-s_{a1}|$, but not $|s_{u1}-s_{w1}|$ and $|s_{v1}-s_{w1}|$. $|\frac{d{\tilde{u}}_{d}^{{}^{\prime }}}{dt}|$ is larger than $h_{2}$ when $|s_{b1}-s_{a1}|=1$. While $|s_{u1}-s_{w1}|$ or $|s_{v1}-s_{w1}|$ is 0, $|\frac{d{\tilde{u}}_{d}^{{}^{\prime }}}{dt}|$ is larger than $h_{2}$ as well. So the fault occurs in $S_{Cn}$. In half a current period, there are instants that $|\frac{d{\tilde{u}}_{d}^{{}^{\prime }}}{dt}|$ is less than $h_{2}$ when $|s_{b1}-s_{a1}|=1$. So the fault is the scaling fault. Figure 13 are the results at 300 km/h and the scaling factor is 1.05 as well. The detailed analysis is omitted.

### 4.3. Fault in Stator Current Sensors

**Figure 14:**
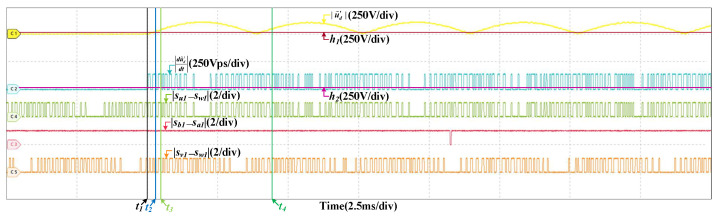
HIL results when the offset fault occurs in $S_{Cu}$ at 200 km/h.

**Figure 15:**
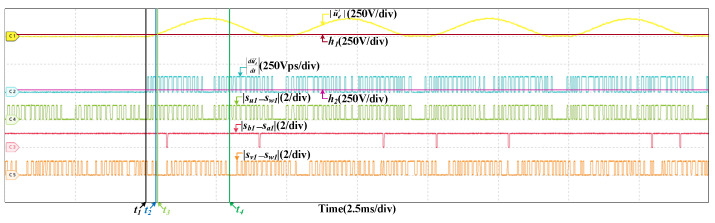
HIL results when the offset fault occurs in $S_{Cu}$ at 300 km/h.

Faults in $S_{Cv}$ are analogous to $S_{Cu}$, take $S_{Cu}$ as an example to show the HIL results when faults occur in stator current sensors. When an offset fault occurs in $S_{Cu}$, The results are shown in Figure 14. The train speed is 200 km/h and the offset value is 10 A. After the fault occurs, $|{\tilde{u}}_{d}^{{}^{\prime }}|$ gets bigger and overs the threshold, the fault is detected. Then, the $|\frac{d{\tilde{u}}_{d}^{{}^{\prime }}}{dt}|$ changes with $|s_{u1}-s_{w1}|$. When $|s_{u1}-s_{w1}|$ is 1, $|\frac{d{\tilde{u}}_{d}^{{}^{\prime }}}{dt}|$ is bigger than $h_{2}$, so the fault does not occur in $S_{Vd}$. There are instants when $|s_{b1}-s_{a1}|$ or $|s_{v1}-s_{w1}|$ is 0 but $|\frac{d{\tilde{u}}_{d}^{{}^{\prime }}}{dt}|$ is larger than $h_{2}$, it can prove the fault does not occur in $S_{Cn}$ and $S_{Cv}$, so the fault is located, which is in $S_{Cu}$. In half a current period, there are no instants when $|s_{u1}-s_{w1}|$ is 1 and $|\frac{d{\tilde{u}}_{d}^{{}^{\prime }}}{dt}|$ is less than $h_{2}$. $|\frac{d{\tilde{u}}_{d}^{{}^{\prime }}}{dt}|$ does not change periodically and the fault type is determined, which is an offset fault. Figure 15 are the results of an offset fault in $S_{Cu}$ at 300 km/h and the offset value is 10 A. The fault diagnosis process is similar and omitted.

**Figure 16:**
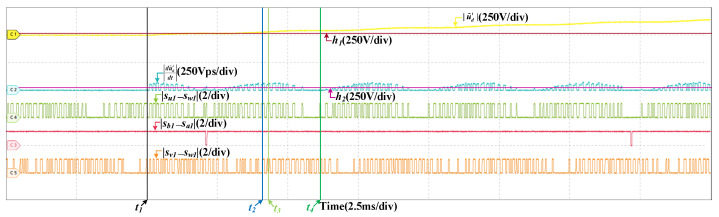
HIL results when the scaling fault occurs in $S_{Cu}$ at 200 km/h.

**Figure 17:**
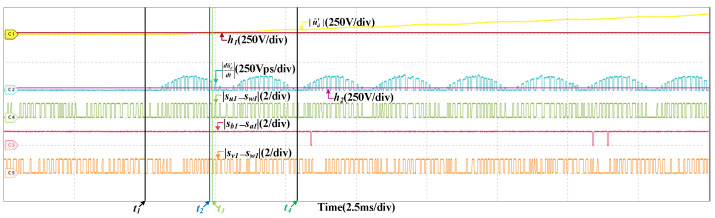
HIL results when the scaling fault occurs in $S_{Cu}$ at 300 km/h.

When a scaling fault occurs in $S_{Cu}$ at the speed of 200 km/h, the scaling factor is 1.05. The results are given in Figure 16. $|\frac{d{\tilde{u}}_{d}^{{}^{\prime }}}{dt}|$ is bigger than $h_{2}$ and the fault is detected. There are instants that $|\frac{d{\tilde{u}}_{d}^{{}^{\prime }}}{dt}|$ is bigger than $h_{2}$, so the fault is not in $S_{Vd}$. When $|s_{b1}-s_{a1}|$ or $|s_{v1}-s_{w1}|$ is 0 but $|\frac{d{\tilde{u}}_{d}^{{}^{\prime }}}{dt}|$ overs $h_{2}$. Once $|s_{u1}-s_{w1}|$ is 0, $|\frac{d{\tilde{u}}_{d}^{{}^{\prime }}}{dt}|$ is less than $h_{2}$. The fault is located, which is in $S_{Cu}$. Then, in half a current period, $|\frac{d{\tilde{u}}_{d}^{{}^{\prime }}}{dt}|$ changes periodically, it has instant when $|s_{u1}-s_{w1}|$ is 1 and $|\frac{d{\tilde{u}}_{d}^{{}^{\prime }}}{dt}|$ is less than $h_{2}$, the fault type is determined, which is a scaling fault. The results of a scaling fault in $S_{Cu}$ at 300 km/h are shown in Figure 17. The scaling factor is 1.05. The detail of fault diagnosis is omitted.

### 4.4. Discussion

There is one point worth discussing. In the fault diagnosis process of current sensors, the command signals are important variables. $|s_{b1}-s_{a1}|$, $|s_{u1}-s_{w1}|$, and $|s_{v1}-s_{w1}|$ are indispensable. The fault location and type identification are realized based on the differences between them. They are PWM (pulse width modulation) signals. If all of them have a high duty cycle or low duty cycle, there will be only a few instants when they are different. For the offset fault, since the differential of residual does not change periodically, once the difference appears, the fault can be located. For the scaling fault, two conditions need to be met, the difference appears and the differential of residual is larger than the threshold. If the differential of residual is less than the threshold when the difference appears, the fault can not be located. However, in most applications, this situation does not happen all the time. The fault can be diagnosed, but the fault diagnosis may cost more time. Moreover, the operating principles of the rectifier and inverter are different, the phases of three phase stator currents are different either. $|s_{b1}-s_{a1}|$, $|s_{u1}-s_{w1}|$, and $|s_{v1}-s_{w1}|$ will not always be the same, which has been proven by the HIL tests. So the proposed fault diagnosis method is suitable for traction converters.

## 5. Conclusions

A sensor fault diagnosis method for the traction converter is proposed in this paper, all of the grid current sensor, DC-link voltage sensor, and stator current sensors are taken into account. The offset and scaling faults are two common and high-incidence faults in sensors, which are considered in this paper. The fault diagnosis method can not only detect and locate the fault, but also identify the fault type. Moreover, there are only two stator current sensors in the system and the faults are incipient. The residual of the DC-link voltage is applied to fault detection and the differentiation of the residual are used to determine the fault location and identify the fault type. First, the residual of the DC-link voltage is calculated, the fault is detected when the residual exceeds the threshold. Then, according to the relationship between the residual and the command signals, the fault can be located. Next, if the the differentiation of the residual changes periodically, the fault will be a scaling fault, otherwise it will be a offset fault. Finally, the HIL platform is established, the HIL tests are carried out in two operation condition, and the test results verify the validity and correctness of the proposed method. 

## Figures and Tables

**Table 1:** Fault diagnosis rules.

Fault Diagnosis Rules	Fault Location	Fault Type
$F_{D}=0$	None	None
$F_{D}=1$; $F_{Vd}=1$;	$S_{Vd}$	Offset fault
$F_{D}=1$; $F_{Cn}=1$; $F_{Cntype}=1$	$S_{Cn}$	Offset fault
$F_{D}=1$; $F_{Cn}=1$; $F_{Cntype}=2$	$S_{Cn}$	Scaling fault
$F_{D}=1$; $F_{Cu}=1$; $F_{Cutype}=1$	$S_{Cu}$	Offset fault
$F_{D}=1$; $F_{Cu}=1$; $F_{Cutype}=2$	$S_{Cu}$	Scaling fault
$F_{D}=1$; $F_{Cv}=1$; $F_{Cvtype}=1$	$S_{Cv}$	Offset fault
$F_{D}=1$; $F_{Cv}=1$; $F_{Cvtype}=2$	$S_{Cv}$	Scaling fault

**Table 2:** Parameters of the system.

Parameter	Symbol	Value
RMS grid voltage	$u_{n}$	1770 V
Traction winding leakage inductor	$L_{n}$	2.3 mH
Traction winding leakage resistor	$R_{n}$	0.068 Ω
DC-link voltage	$u_{d}$	3000 V
Support Capacitor	$C_{d}$	3 mF
Stator resistance	$R_{s}$	0.1065 Ω
Stator inductance	$L_{s}$	1.318 mH
Rotor resistance	$R_{r}$	0.0663 Ω
Rotor inductance	$L_{r}$	1.93 mH
Mutual inductance	$L_{m}$	53.6 mH
Rated voltage	$U_{rate}$	2750 V
Rated speed	$n_{rate}$	4100 r/min
Rated frequency	$f_{rate}$	138 Hz
Rated output power	$P_{rate}$	562 kW
Rated slip frequency	$s_{rate}$	0.04
Number of the pole pairs	$n_{p}$	2

## Data Availability

Not applicable.
